# The Analgesic Mismanagement of a Patient With Ehlers-Danlos Syndrome (Hypermobility Variant): A Case Report

**DOI:** 10.7759/cureus.45713

**Published:** 2023-09-21

**Authors:** Tharajan Gunendran, Namitha Uma Dwarakanath

**Affiliations:** 1 Medicine, All Saints University, Roseau, DMA; 2 Medicine, Tbilisi State Medical University, Tbilisi, GEO

**Keywords:** ehlers-danlos syndrome hypermobility type (eds-ht), ehlers-danlos syndrome, morphine milligram equivalents, mechanical pain, nociceptive pain, analgesic consumption, who analgesic ladder, mme, multi-modality pain management, opiate misuse

## Abstract

Ehlers-Danlos syndrome (EDS) is a rare disorder affecting the connective tissue, resulting in joint hypermobility, elastic skin, and often chronic pain, especially in the hypermobility variant. Although opioids are commonly prescribed for pain, they can lead to opioid use disorder (OUD) and overdose. A 67-year-old female with Ehlers-Danlos syndrome hypermobility type (EDS-HT), osteoarthritis (OA), and anxiety received opioid-based pain management for a decade before changing her primary care physician. Her medications included oxycodone and morphine sulfate extended-release (ER) at different dosages. To lower overdose risk, her morphine milligram equivalents (MME) were tracked, and a step-by-step opioid tapering process was started. Diagnosing EDS is difficult due to symptom overlap with other connective tissue disorders. Chronic pain in EDS involves both nociceptive and neuropathic pain, necessitating a comprehensive pain management approach. The essential components of pain management include non-opioid medications, physical therapy, and psychological support. Opioids should be used cautiously in EDS patients because of connective tissue vulnerabilities and potential side effects. Personalized plans for opioid tapering may be appropriate for those on long-term opioid therapy. Managing EDS-related chronic pain requires a tailored, multidisciplinary approach. Early and accurate diagnosis and specialized healthcare providers familiar with EDS are crucial for effective pain management. Ongoing research and evidence-based pain management approaches are vital to address the unique needs of EDS patients, promoting better pain relief and overall well-being. Through meticulous evaluation and personalized treatment plans, healthcare professionals can better support EDS patients in managing chronic pain and reducing opioid dependence and misuse risks. A comprehensive approach, incorporating non-opioid medications, physical therapy, and psychological support, can offer effective pain relief and improve the quality of life for those living with EDS.

## Introduction

Ehlers-Danlos syndrome (EDS) is a rare syndrome affecting connective tissue. It is characterized by abnormalities in collagen metabolism, resulting in deficiencies and disordered deposition of collagen [1-3]. A health issue that mainly affects the skin and joints can result in constant physical discomfort, often accompanied by emotional stress. Ehlers-Danlos syndrome hypermobility type (EDS-HT) is a specific type that can be particularly challenging, as musculoskeletal pain becomes a significant aspect of patients' physical symptoms. Pain related to EDS usually appears as recurring joint pain caused by joint instability, such as sprains and dislocations, but it can eventually lead to widespread myalgias, arthralgias, and neuropathic symptoms. It is recommended by the Centers for Disease Control and Prevention (CDC) to evaluate opioid dosage using morphine milligram equivalents (MME) per day metrics. Dosages that exceed 50 MME/day are not advised because of the limited pain or functional benefits they provide and the increased risk of overdose [4-6]. To manage pain effectively, it is important to understand its type and use specific treatments. Nociceptive pain can be eased with anti-inflammatory therapies, while neuropathic pain may require antidepressants, antiepileptics, or opioids for relief [7-10]. The increasing incidence of prescription opioid use disorders (OUDs) and opiate-related overdose deaths is a significant cause for worry. The proper awareness and diagnosis of EDS can greatly benefit patients by improving their self-esteem, reducing confusion about physical sensations, and implementing more effective pain management strategies. Healthcare professionals can better support individuals with EDS by carefully evaluating and tailoring pain management approaches, which can promote safer opioid use practices [11-16]. The guidelines for tapering opioids suggest lowering the dosage for patients receiving chronic opioid therapy (COT) to reduce potential dangers such as misuse and overdose [17,18].

## Case presentation

This report explains how we managed chronic pain in a 67-year-old female patient diagnosed with Ehlers-Danlos syndrome (EDS). Our team reviewed her medical records, which included information from her primary care physician and previous healthcare providers. This helped us gain an understanding of her medical history and treatment journey. The patient had been diagnosed with several medical conditions such as EDS, chronic pain syndrome, muscle spasms, osteoarthritis (OA), repetitive sprain syndrome, idiopathic peripheral nerve neuropathy, anxiety, and depression. She had received pain management from a pain management specialist and primary care provider for over a decade before switching to her current primary care physician in 2022. According to her medical notes, her former primary care provider felt "overwhelmed" by her EDS chronic conditions, which was why she sought other treatments. Over time, the patient tried different methods such as activity modification, physical therapy, medication, and therapeutic injections, but none produced the desired results.

During her first appointment with her current primary care physician, the patient presented a list of medications that included oxycodone hydrochloride (HCl) 30 mg taken orally four times daily (po QID), morphine extended-release (ER) 30 mg taken orally four times daily (po QID), gabapentin 300 mg taken orally four times daily as needed (po QID PRN), diazepam 10 mg taken orally twice daily (po BID), losartan 100 mg taken orally once a day (po QD), and amlodipine 5 mg taken orally once a day (po QD). She confirmed that she was taking her medications as prescribed and had not experienced any adverse effects. In 2020, her analgesic regimen was adjusted to include oxycodone hydrochloride (HCl) 30 mg taken orally five times daily and morphine sulfate ER 30 mg taken orally twice daily (po BID), resulting in a morphine milligram equivalent (MME) score of 285/day. By 2021, her medication was modified to oxycodone hydrochloride (HCl) 30 mg taken orally four times daily and morphine sulfate ER 30 mg taken orally twice daily (po BID), resulting in an MME score of 240/day. However, her morphine sulfate ER dosage was gradually increased to 60 mg taken orally twice daily, resulting in an MME score of 300/day. In early 2022, her morphine sulfate ER dosage was tapered back down to 30 mg taken orally twice daily, resulting in an MME score of 240/day.

When the patient began receiving pain management care from her new primary care physician, she was required to sign a patient responsibility agreement for controlled substance prescription. The treatment plan included monthly, bimonthly, or trimonthly in-person office visits, as determined by the prescribing physician. During each appointment, the patient underwent an Alcohol Use Disorders Identification Test (AUDIT) and Drug Abuse Screening Test (DAST). At her first visit in 2022, the patient was informed that her MME score was 240/day, indicating an 8.9 times higher risk of overdose due to her substantial use of opioids. The primary focus of her care was to manage potential overdose risks and address her pain symptoms. To achieve this, the healthcare provider implemented a stepwise approach to opioid tapering. The patient's morphine sulfate dosage was gradually lowered from 30 mg twice daily, decreasing MME from 240 to 180 daily. Weekly reductions of 5%-10% were implemented. During a 2022 checkup, the patient reported anxiety and depression due to pain. Patient Health Questionnaire-9 (PHQ-9) and Generalized Anxiety Disorder-7 (GAD-7) scores confirmed this. The patient was prescribed fluoxetine 20 mg to address these issues. The patient received cognitive behavioral therapy (CBT) for emotional well-being and had her pain management plan adjusted due to increased back pain with radiculopathies. Fluoxetine was replaced with venlafaxine ER 37.5 mg, known for treating neuropathic pain. Diazepam was gradually reduced to 5 mg po BID, while venlafaxine was increased to 75 mg for effective treatment. The MRI showed bulges and nerve compression in the lower back. To match the patient's preferences and past success with morphine, a combination of oxycodone and morphine sulfate ER was prescribed. Oxycodone was gradually reduced to three times daily, while morphine sulfate ER 30 mg was reintroduced. The healthcare provider reduced the patient's oxycodone dosage gradually to lower MME per day while maintaining pain relief. However, the patient reported more pain, sleep difficulties, and urinary issues. The healthcare provider prescribed antibiotics and escitalopram 20 mg to manage anxiety, depression, and insomnia. The patient's preferences were respected, and a gradual tapering method was used to wean off opioids.

## Discussion

The clinical diagnosis of hypermobile Ehlers-Danlos syndrome (hEDS) involves three major criteria that must be met simultaneously: generalized joint hypermobility, musculoskeletal complications, and/or family history [1]. The Beighton score is one of the primary tools utilized for diagnosing generalized joint hypermobility in EDS, which evaluates the range of motion in particular joints. The Beighton score grants one point for each of five maneuvers, including passive dorsiflexion of each fifth finger greater than 90°, passive apposition of each thumb to the flexor surface of the forearm, hyperextension of each elbow greater than 10°, hyperextension of each knee greater than 10°, and the ability to place the palms flat on the floor with the knees fully extended. A score of ≥5 indicates generalized joint hypermobility. In addition, there is a clinical overlap between the subtypes of EDS and other connective tissue disorders. This overlap can cause confusion during diagnosis, resulting in delayed or incorrect diagnosis. This can impact the timely implementation of effective pain management strategies [2].

Table 1 outlines the potential differential diagnoses to consider when evaluating a case suspected to be hEDS. Chronic pain is a common and debilitating symptom for those with hEDS. Often felt as muscular or myofascial discomfort localized around or between joints, this pain may be linked to myofascial spasms. Tender points consistent with fibromyalgia can often be detected, particularly in the paravertebral musculature. Myofascial spasms are suggested to occur in response to chronic joint instability [1,2]. Many EDS patients suffer from chronic pain, which often leads to the use of opioids for pain management. While opioids can provide effective short-term relief, their long-term use carries significant risks, such as the potential for dependence, tolerance, and opioid use disorder (OUD). Studies have shown that patients who use opioids for chronic non-cancer pain are at higher risk of developing OUD, and the length of time they use opioids is a key factor in determining that risk [3]. The guidelines set forth by the CDC stress the importance of being cautious when prescribing opioids. The main goal is to decrease the likelihood of misuse, abuse, addiction, and overdose [4]. Although opiates can effectively relieve moderate to severe pain, their use in patients with EDS must be carefully considered. The long-term use of opioids can cause tolerance and hyperalgesia, which may increase the chances of developing OUD. A study revealed that 41% of EDS patients who were treated with opiates for pain management experienced negative reactions related to opioids, and 19% became dependent on opioids [5].

**Table 1:** Differential Diagnoses of Hypermobile Ehlers-Danlos Syndrome (hEDS): Symptomatic Similarities and Distinctive Features

Differential Diagnoses	Similarities to hEDS	Distinctive Characteristics
Marfan syndrome	Joint hypermobility and skin elasticity	Eye abnormalities and aortic enlargement
Osteogenesis imperfecta	Joint hypermobility and ecchymosis	Bone fragility and blue sclerae
Loeys-Dietz syndrome	Joint hypermobility and skin translucency	Vascular complications and arterial aneurysms
Stickler syndrome	Joint hypermobility and musculoskeletal pain	Primarily musculoskeletal manifestations
Lupus	Joint pain and skin manifestations	Renal, hematologic, and neurological symptoms and malar rash
Fibromyalgia	Chronic pain and fatigue	Widespread pain, sleep disturbances, and headaches
Polymyalgia rheumatica	Muscle stiffness and joint pain	Affected individuals >50 years old and shoulder and hip stiffness
Hyperthyroidism	Muscle weakness and fatigue	Weight loss and heat intolerance
Sjögren's syndrome	Joint pain and fatigue	Xerostomia, xerophthalmia, and renal and lung involvement
Joint hypermobility syndrome (JHS)	Joint hypermobility and musculoskeletal pain	Primarily musculoskeletal manifestations
Nutritional deficiencies (scurvy)	Easy bruising and joint pain	Scurvy: swollen, bleeding gums and anemia

**Figure 1:**
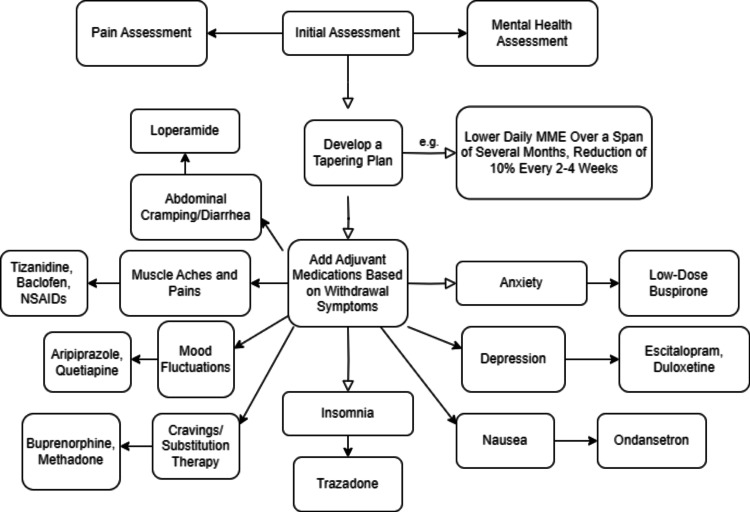
Comprehensive Opioid Tapering Strategy Incorporating Adjuvant Medications MME, morphine milligram equivalent; NSAIDs, nonsteroidal anti-inflammatory drugs

Figure 1 illustrates a structured approach to opioid tapering, incorporating the use of adjuvant medications to manage potential withdrawal symptoms. It is important to consider alternative pain management techniques for long-term relief. Non-opioid medications, such as nonsteroidal anti-inflammatory drug (NSAIDs), acetaminophen, anticonvulsants, and antidepressants, have proven to be effective in managing pain and improving function. NSAIDs can help reduce inflammation and ease musculoskeletal pain, while acetaminophen is helpful for mild to moderate pain. Anticonvulsants such as gabapentin and pregabalin are effective for neuropathic pain and can be beneficial for EDS patients experiencing neuropathic pain symptoms. Tricyclic antidepressants and serotonin-norepinephrine reuptake inhibitors (SNRIs) have also proven to be effective in managing neuropathic pain and may be a part of the pain management strategy [6]. There is a lack of clear evidence about the most effective rehabilitative settings or physiotherapy treatments for hEDS due to the absence of randomized controlled trials. Nevertheless, some studies have shown positive outcomes for patients treated with physiotherapy, with 63.4% experiencing improvements in pain and overall health. Physical and occupational therapy can help strengthen muscles, stabilize joints, and improve flexibility, reducing strain on joints and the risk of injuries [8]. Opioids may induce sedation, constipation, nausea, and respiratory depression, among other adverse reactions. EDS patients who already have autonomic dysfunction and gastrointestinal problems may experience worsened symptoms due to opioid-induced constipation. Additionally, individuals with chronic pain in EDS may develop tolerance and dependence, necessitating higher opioid doses over time. This increases the risk of negative effects and the development of OUD [9,10].

The cause of pain in EDS is complicated and involves both nociceptive and neuropathic pain pathways. Nociceptive pain is caused by pain receptors being activated in response to tissue damage or inflammation. In EDS, this type of pain can occur due to joint instability, microtrauma, and repeated subluxations or dislocations, which can damage ligaments, tendons, and joints. Neuropathic pain, on the other hand, is caused by damage or dysfunction to the nervous system, which leads to abnormal processing of pain signals [11]. In EDS, this type of pain can be caused by nerve subluxations, nerve compression, and axonal neuropathies due to connective tissue defects affecting nerve structures. Additionally, central sensitization, a condition where the central nervous system becomes hypersensitive to pain signals, resulting in increased pain perception and generalized hyperalgesia, may also be present. This may be due to the continuous stimulation of peripheral nociceptors by mediators released by the aberrant extracellular matrix (ECM) in EDS. The interplay of these nociceptive and neuropathic pain mechanisms contributes to the complexity and variability of pain experienced by individuals with EDS. Therefore, a comprehensive approach to pain management that targets both nociceptive and neuropathic components is necessary to alleviate pain effectively in EDS patients.

Studies suggest that people with hypermobile EDS or joint hypermobility syndrome may exhibit increased windup to repeated stimuli and decreased exercise-induced analgesia, which supports the presence of central sensitization in these individuals. Fibromyalgia, a widespread musculoskeletal pain syndrome often associated with central sensitization, occurs in 42% of adults with EDS [11,12]. Although opiates are effective in relieving moderate to severe pain, caution should be exercised when prescribing them to patients with EDS due to the condition's connective tissue vulnerabilities, as well as the risk of side effects and complications. Opioids interact with the mu opioid receptors in the brain's reward system, triggering the release of dopamine and intense feelings of pleasure. However, the chronic use of opioids can lead to tolerance and hyperalgesia, where patients require higher doses for the same level of pain relief and become more sensitive to pain over time [13,14]. Drug overdoses, particularly from opioids, have become a major public health issue. In 2017, the Centers for Disease Control and Prevention (CDC) reported over 70,000 drug overdose deaths, with 68% of those attributed to opioids. Additionally, synthetic opioids were involved in nearly 60% of all opioid-related overdose fatalities. To address the complex pain experienced by patients with EDS, a comprehensive approach that incorporates various fields such as pain management, rheumatology, physical therapy, and psychology is essential [15]. The CDC recommends that opioids should only be used for chronic pain when other treatments have been considered and the potential benefits and risks have been fully assessed. It is recommended that healthcare providers begin with the lowest effective dose and avoid increasing it beyond 50 MME/day, as higher doses have been linked to higher risks.

**Figure 2:**
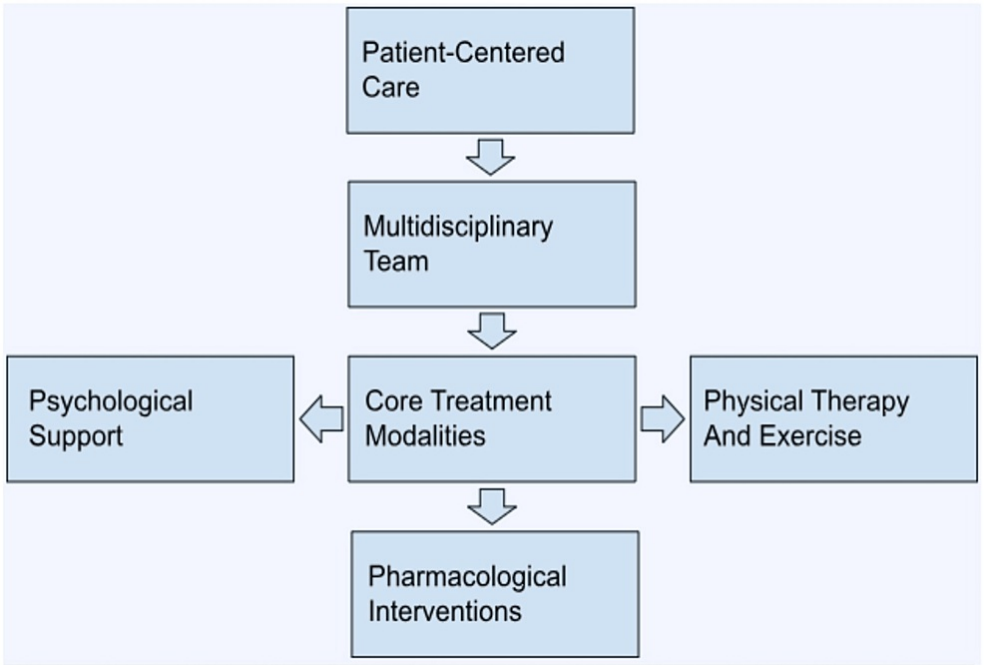
Comprehensive Pain Management Approach for EDS EDS: Ehlers-Danlos syndrome

Figure 2 illustrates a comprehensive pain management approach for individuals with Ehlers-Danlos syndrome (EDS). This model emphasizes patient-centered care, a multidisciplinary team, core treatment modalities, and shared decision-making to effectively address the complexities of chronic pain in EDS. When considering adjustments to dosage, prescribers should evaluate the individual benefits and risks, discuss treatment goals with patients, and consider other effective pain management strategies [16,17]. Opioid tapering, which involves gradually reducing opioid dosages, can be considered for those taking opioids in the long term. However, a retrospective study found that undergoing an opioid taper was associated with over four times higher odds of care termination in the following year compared to continuing the same opioid dose. The improper tapering or discontinuation of opioids can lead to withdrawal symptoms and increased pain. Therefore, healthcare providers should work collaboratively with their patients to create personalized tapering plans that address their specific concerns and needs. This approach involves gradually decreasing opioid dosages to ensure the patient's safety and well-being [18].

## Conclusions

People with Ehlers-Danlos syndrome (EDS) have a group of heritable connective tissue disorders (HCTDs) that cause joint hypermobility, skin hyperextensibility, and tissue fragility. Chronic pain is a common and debilitating symptom for those with EDS. The pain's complexity is due to both nociceptive and neuropathic pain mechanisms. While opioids may provide short-term relief, their long-term use for chronic pain management comes with risks. Therefore, there is a need for a comprehensive and multidisciplinary approach to pain management. Non-opioid medications, physical therapy, and psychological support are all important in improving pain outcomes for people with EDS. Early and accurate diagnosis, along with specialized healthcare providers familiar with EDS, is crucial in facilitating effective pain management and improving the quality of life for those living with this challenging condition. To address the unique needs of people with EDS and promote better pain relief and overall well-being, continued research and evidence-based approaches to pain management are essential.
